# Prosthetic Dentistry

**Published:** 1893-10

**Authors:** 

**Affiliations:** Chicago.


ARTICLE V.

### PROSTHETIC DENTISTRY.

BY DR. HASKELL, CHICAGO.

In 1845 I commenced the study of dentistry with a practicing dentist in Boston. My first beginning was the grinding of material for the making of porcelain teeth. Perhaps all of you are aware that porcelain teeth were made of feldspar, quartz, and clay, ground in a quartz mortar. I have spent months grinding material and mixing material, and passed years among other things grinding block teeth, such as are now represented by gum sections, made in molds. In those days teeth were made for each case. All were metal plates, gold, platinum, and the teeth were carved for those who used them. The dentist would send the swaged plate with the wax, indicating length and thickness of the teeth. The material used was soft and had to be handled with care. The teeth were all carved out, six in the front block and four in the posterior. Pins were soldered in the teeth and blocks fastened to the teeth. Afterwards the teeth were made with the pins set down into them and soldered, just the same as all plate teeth nowadays. In those days they were soldered to the plate. You who have been in practice only since the introduction of rubber know nothing of what this meant. The dentist had to select a set of single gum teeth, grind them to the plate, articulate them, invest and solder. We did not use gas in those days, but alcohol. We had to solder up this full set, upper and lower, with alcohol. You may congratulate yourselves that you are living and working in this age of dentistry.

On the introduction of rubber, in about the year 1858, the first rubber plates were made. I was then in Chicago with Dr. Allport. We commenced using this rubber soon after it was introduced.

I wish to say right here that on the introduction of rubber there was a retrograde movement in mechanical dentistry, which did not change up to a very recent date. I may say that the forward movement began when crown- and bridge-work was introduced, from the fact that it demanded of dentists a knowledge of the use and working of gold. From that time there began to be an advance movement, because, as I said, every dentist needed to know how to work gold, and that was an inducement to him not only to make his crown- and bridge-work, but also metal plates.

One great difficulty with prosthetic dentists is the teaching of our colleges. You take nearly all of our graduates and they are better prepared to fill a tooth than to make an artificial denture. With all the studies which the dental student has to take up—a large portion of the studies of the medical student, and special studies for dental students, then the time he has left to spend on the really practical part of his college education is small. The student spends more time in the theories; he is more interested in operative than in prosthetic dentistry. The student is partly to blame, but with the present method of instruction in colleges I do not see how it is possible for the student to make that progress in technical instruction in the laboratory that ought to be made. It cannot be done in the lecture-room, though there must be lectures on general theories. From my experience in different dental colleges, I have had pretty good opportunities to know what I am talking about in this matter. There must be some lectures on studies on general theories; but it is just a waste of time for a lecturer to undertake to describe a method that the student has got to follow in the laboratory. It goes in at one ear and out of the other. He never understands it till he does it himself.

There is a great deal of time wasted in the lecture-room. The student had better be in the laboratory. Another great difficulty is the fact that many of the demonstrators in our colleges are young, inexperienced men. You will often find a last year's graduate as demonstrator. The demonstrator in the laboratory should be a man of experience. The lecturer ought to go from the lecture-room to the laboratory, and follow up his lectures by personal instruction, so students may understand what they have been taught in the lecture-room. I do not know how to remedy this matter, except it be to relegate the practical part to the last month or two of the term, putting the students right into the laboratory and keeping them there, lectures and all. They will learn more in this way than in the whole course of two or three years. The difficulty is that students are so apt to be graduates on theory. I have seen dental students who could tell you the anatomy of the foot, but could not make a rubber plate. This is all wrong, and was one of my chief reasons for establishing a post-graduate school.

I have a large number of models—all metal plates. I have three long shelves filled with models of full upper cases. Till a dentist can take in at a glance the peculiarities of these, he does not begin to realize the condition of the human jaw, after wearing a plate more or less years. I have cases that show a small amount of absorption where the teeth have been out for years. Most of the ridges are small. I do not think most dentists have had an opportunity to see a collection of models arranged like this.

This condition of things did not exist before the introduction of rubber plates. Within a very few years after I commenced the use of rubber, I began to notice a great change in the alveolar ridge. It grew on us constantly. I could not account for it.

After I had been in Chicago about fifteen years, I returned to Boston and spent a couple of months there, doing work for old and new patients. I took occasion to examine many mouths, among them patients who had worn plates from fifteen to twenty-five years. The gums under those plates did not seem to have changed at all. I was astonished. I did expect to find considerable change.

Last September I was in Boston. I saw one of the first continuous gum plates I had ever made. It had never been repaired. Another was a gold plate; after forty years, in splendid condition. Facts like these mean something. Theories are very well.

Here Prof. Haskell showed models of different cases, of such interesting nature and great variety that it would certainly profit any practitioner to study them well.

There is nothing that has so simplified the fitting of rubber plates as Babbitt metal. There is no shrinkage. It is hard, yet sufficiently soft and smooth. You can use your oiled sand successfully.

Dr. Billings has been doing a valuable service to the profession in the last few years, in introducing his prepared sand for dental work. I am glad to know that his efforts are appreciated.

In making plates, there is one thing overlooked, and largely responsible for the effect on appearance of upper lip. You can raise the impression at the base for the extension of the cuspid teeth. It is very simple. We have an invariable rule: There is not a solitary case where there is not loss of the cuspid eminence, if the cuspids have been extracted one year or more. The plate should be made fuller and higher over the cuspid teeth. The fullness should not come under the nose.

There are some peculiarities of the human jaw. In ninety-five out of one hundred mouths there is a depression on the left side of the jaw, over the cuspid tooth; not on the right side. Another thing—the process is shorter on the left than on the right side. The reason we so often see artificial teeth too short on the right side is because the dentist arranged the cutting teeth according to his model, instead of by the mouth. What is the cause of this? I can hardly find a dentist who has even noticed the fact. I had a theory! That most people put the food into their mouths at the right side, in the region of the cuspid tooth. If the teeth are sufficiently good, the food is masticated more on the right side, consequently it is developed more on the right side than on the left.

Dr. Talbot's theory is this, and it will surprise you all;

Are you aware of the fact that in chewing, your jaw moves from right to left, not from left to right? Gradually, in course of time, the process and teeth are changed.

Oftentimes, on the lower jaw one side of the jaw stands farther from the median line than the other—the left side. I do not understand why this is. On the left side of the jaw you will often find that the teeth are longer and fuller than on the right.

I use no gum sections. I use plain teeth, because I cannot use gum sections satisfactorily, their arrangement being so arbitrary. There are more failures from faulty articulation than from all other causes combined. Everything is right until the patient closes the jaw.

The cutting edges of the six anterior teeth should form part of a circle, of which the radius is the width of three anterior teeth at one side of the median line. This circle should pass about through the middle of the grinding surface of the second bicuspids, and through the middle of the posterior lingual cusp of the first.

I think aluminum, as it is now manufactured pure, is an excellent substitute for rubber. About twenty-two gage is very nice to work. It is easily swaged, and preferable to rubber.

I do not condemn rubber; many cannot afford anything else.—*Items of Interest.*

